# Optimism

**Published:** 1892-01

**Authors:** Earnest Hawthorne


### OPTIMISM.

BY EARNEST HAWTHORNE.

Were we to know what blessed rest awaits,
Impatient might we grow of flinty ways;
The unveiled Light Eterne would only daze
Earth focused eyes. Angels were no fit mates
For mortal men. In love, not scorn, the Fates
Have sealed our eyes, that our appointed days
On earth well may be spent; and blame, not praise,
Be theirs who—fond, rebellious, rash ingrates—
Chafe at the limits which are man's defense.
Children, what manhood means we cannot know,
And need not, if we could. To learn; to grow,
By earthly joy, pain, labor, rest—through sense
To blossom into soul—is given us breath.
Who truly lives, nor dreads nor longs for death.

